# Supplementary material for: A comparison of embedding aggregation strategies in drug–target interaction prediction
Source: BMC Bioinformatics. 2024 Feb 6;25:59. doi: 10.1186/s12859-024-05684-y (PMC10845509; doi:10.1186/s12859-024-05684-y)
Supplement: Supplementary file 1 — Additional file 1: A) Contains theorems that show how all three aggregation strategies we consider are universal approximators. B) Contains detailed information about the hyperparameter ranges of every experiment included in this work. [file 12859_2024_5684_MOESM1_ESM.pdf]

Appendix A Universal approximation

In this appendix we formally prove that the three aggregation strategies are universal approximators, using existing mathematical results [39, 40] as building blocks. To this end, let us first formally introduce hypothesis spaces for the protein and compound branches. For simplicity, we restrict our analysis to $\mathcal{X} = \mathbb{R}^{K_1}$ and $\mathcal{T} = \mathbb{R}^{K_2}$ with $K_1, K_2 \in \mathbb{N}$, but extensions to other types of feature spaces could be easily made. For the protein branch, we will show particular interest in the hypothesis space $\mathcal{H}_{\mathcal{X},\text{MLP}}$ consisting of functions of the form

$$\vec{g}(\vec{x}) = \vec{C}^{(1)}(\sigma \circ (\vec{B}^{(1)}\vec{x} + \vec{b}^{(1)}))\,,$$

with parameterization $\vec{\theta}_{\mathcal{X},\text{MLP}} = (\vec{b}^{(1)}, \vec{B}^{(1)}, \vec{C}^{(1)}, L_1, D_1)$, where $\vec{b}^{(1)} \in \mathbb{R}^{1\times K_1}$, $\vec{B}^{(1)} \in \mathbb{R}^{L_1 \times K_1}$, $\vec{C}^{(2)} \in \mathbb{R}^{D_1 \times L_1}$ and $K_1, L_1, D_1 \in \mathbb{N}$. Furthermore, $\sigma\circ$ represents the sigmoid activation function.

Similarly, for the compound branch we define the hypothesis space $\mathcal{H}_{\mathcal{T},\text{MLP}}$ consisting of functions of the form

$$\vec{h}(\vec{t}) = \vec{C}^{(2)}(\sigma \circ (\vec{B}^{(2)}\vec{t} + \vec{b}^{(2)}))\,,$$

with parameterization $\vec{\theta}_{\mathcal{T},\text{MLP}} = (\vec{b}^{(2)}, \vec{B}^{(2)}, \vec{C}^{(2)}, L_2, D_2)$, where $\vec{b}^{(2)} \in \mathbb{R}^{1\times K_2}$, $\vec{B}^{(2)} \in \mathbb{R}^{L_2 \times K_2}$, $\vec{C}^{(2)} \in \mathbb{R}^{D_2 \times L_2}$ and $K_2, L_2, D_2 \in \mathbb{N}$.

Let us first present a theorem that establishes universal approximation for the protein and compound branches. The proof of this theorem is an immediate generalization of a classical proof given by [39] for the case $D_1 = 1$ or $D_2 = 1$.

Theorem 1. *Let $\mathcal{C}(\mathcal{X})$ and $\mathcal{C}(\mathcal{T})$ denote the sets of all continuous functions $\vec{g} : \mathcal{X} \to \mathbb{R}^{D_1}$, for any $D_1 \in \mathbb{N}$, and $\vec{h} : \mathcal{T} \to \mathbb{R}^{D_2}$, for any $D_2 \in \mathbb{N}$. Then, $\mathcal{H}_{\mathcal{X},MLP}$ and $\mathcal{H}_{\mathcal{T},MLP}$ are universal approximators of $\mathcal{C}(\mathcal{X})$ and $\mathcal{C}(\mathcal{T})$, respectively:*

(i) For all $\vec{g} \in \mathcal{C}(\mathcal{X})$ and $\epsilon > 0$ there exists $\vec{g}' \in \mathcal{H}_{\mathcal{X},MLP}$ such that

$$\sup_{\vec{x}\in\mathcal{X}} \parallel \vec{g}(\vec{x}) - \vec{g}'(\vec{x}) \parallel < \epsilon\,.$$

(ii) For all $\vec{h} \in \mathcal{C}(\mathcal{T})$ and $\epsilon > 0$ there exists $\vec{h}' \in \mathcal{H}_{\mathcal{T},MLP}$ such that

$$\sup_{\vec{t}\in\mathcal{T}} \parallel \vec{h}(\vec{t}) - \vec{h}'(\vec{t}) \parallel < \epsilon\,.$$

Universal approximation capabilities of the three types of aggregation strategies then immediately follows from existing mathematical results. To show this formally, let us first define universal approximation for DTI prediction.

Definition 1. *Let $\mathcal{C}(\mathcal{X} \times \mathcal{T})$ be the space of all continuous functions of type $f : \mathcal{X} \times \mathcal{T} \to \mathbb{R}$. A hypothesis space $\mathcal{H}$ is a universal approximator of $\mathcal{C}(\mathcal{X} \times \mathcal{T})$ if for all*

$f \in \mathcal{C}(\mathcal{X} \times \mathcal{T})$ and $\epsilon > 0$ there exists $f' \in \mathcal{H}$ such that

$$\sup_{\vec{x}\in\mathcal{X},\vec{t}\in\mathcal{T}} |f(\vec{x},\vec{t}) - f'(\vec{x},\vec{t})| < \epsilon\,.$$

Let us now consider the hypothesis spaces $\mathcal{H}_{\text{MLP}}$, $\mathcal{H}_{\text{DP}}$, $\mathcal{H}_{\text{TP}}$ introduced in Section 2. We show that all three of them are universal approximators.

Theorem 2. *If $\mathcal{H}_{\mathcal{X}}$ and $\mathcal{H}_{\mathcal{T}}$ are universal approximators of $\mathcal{C}(\mathcal{X})$ and $\mathcal{C}(\mathcal{T})$, respectively, then $\mathcal{H}_{\text{DP}}$, $\mathcal{H}_{\text{MLP}}$ and $\mathcal{H}_{\text{TP}}$ are universal approximators of $\mathcal{C}(\mathcal{X} \times \mathcal{T})$.*

Proof. As discussed in [39], the universality of $\mathcal{H}_{\text{DP}}$ follows from the Stone–Weierstras theorem. The authors assume that $\mathcal{X}$ and $\mathcal{T}$ are Hausdorff spaces, but here we limit our analysis to Euclidean spaces for $\mathcal{X}$ and $\mathcal{T}$. The universality of $\mathcal{H}_{\text{TP}}$ follows from the fact that a dot product can be rewritten as a tensor product. To this end, in Eq. **??**, we choose $D = D_1 = D_2$, $w_{kl} = 1$ when $k = l$ and $w_{kl} = 0$ when $k \neq l$. Similarly, the universality of $\mathcal{H}_{\text{MLP}}$ immediately follows from the fact that a dot product, like any other continuous function of several variables, can be uniformly approximated by a multi-layer perceptron. □

Appendix B Hyper-parameter ranges

In the following section, we detail the hyperparameter space we explore:

- MLP strategy:
  - layers: [1, 2, 3]
  - nodes per layer: [4, 8, 16, 32, 64, 128, 256, 512, 1024, 2048]

and the three input branches:

- MLP compound or protein branch:
  - layers: [1, 2, 3, 4]
  - nodes per layer: [4, 8, 16, 32, 64, 128, 256, 512, 1024, 2048]
- CNN compound or protein branch:
  - layers: [1, 2, 3, 4]
  - filters per layer: [16, 32, 64, 128]
  - kernels per layer: [4, 8, 12, 16]
- MPNN compound branch:
  - depth: [1, 2, 3]

Finally, the range for both the compound and protein embedding was the following:

$$[2, 3, 4, 8, 16, 32, 64, 128, 256, 512]$$
